# Secondary Abutment Syndromes of the Wrist in Trauma: A Pictorial Essay

**DOI:** 10.5334/jbsr.1558

**Published:** 2018-08-30

**Authors:** Marc Mespreuve, Karl Waked, Koenraad Verstraete

**Affiliations:** 1 Department of Radiology, St.-Maarten General Hospital, Leopoldstraat 2, 2800 Mechelen, BE; 2 Department of Radiology, Ghent University, De Pintelaan 185, 9000 Ghent, BE; 3 Department of Plastic Surgery, Brussels University, Laarbeeklaan 101, 1090 Brussels, BE

**Keywords:** wrist, abutment, trauma, radiographs, MRI

## Abstract

Traumatic lesions of the wrist occur frequently and may give rise to underdiagnosed secondary abutment syndromes. The latter are a common cause of incapacitating pain and limited range of motion, despite minimal or even absent alterations on radiographs. Moreover, the complex wrist anatomy often results in ignorance or underappreciation of these syndromes.

This paper presents a pictorial review of frequent and rare secondary abutment syndromes at the wrist joint, which – in contrast to primary abutment syndromes – are not based on anatomical variants or congenital deformations. The merit of each imaging modality is briefly mentioned.

## Introduction

Traumatic wrist lesions occur frequently. Subsequently, secondary abutment syndromes (SAS), a common cause of incapacitating pain and limited range of motion in spite of minimal or absent alterations on radiographs, may arise. They are often underappreciated due to the complex wrist anatomy and call for a thorough analysis of all wrist components.

The aim of this pictorial review is to present an overview of SAS and to highlight the role of imaging.

## Normal anatomy

The wrist is a complex structure of cartilaginous joints with little intrinsic stability, but mainly relies on soft tissue constraints from various ligaments. The three-dimensional motion is very susceptible to disturbances of their complex surfaces and to ligamentous lesions [1].

## Biomechanics and pathology

Wrist fractures are frequently missed on radiographs [2]. Ligamentous lesions [3] are even more susceptible to false negative interpretations. Both may be the cause of misalignment, pathological mobility or instability.

The secondary repetitive bony impaction may result in contusion [4] with the development of subchondral bone marrow oedema (BMO), opposing articular surfaces chondromalacia, subchondral cyst formation, and surrounding synovitis.

## Clinical manifestation

SAS may give rise to complaints, sometimes appearing years after trauma. The predominant symptoms are restricted motion and incapacitating pain, exacerbated by activity. SAS may have a negative impact on the three-dimensional hand positioning during daily activities [5].

## Pathology and imaging

### Radioscafoid abutment

**Figure 1:**
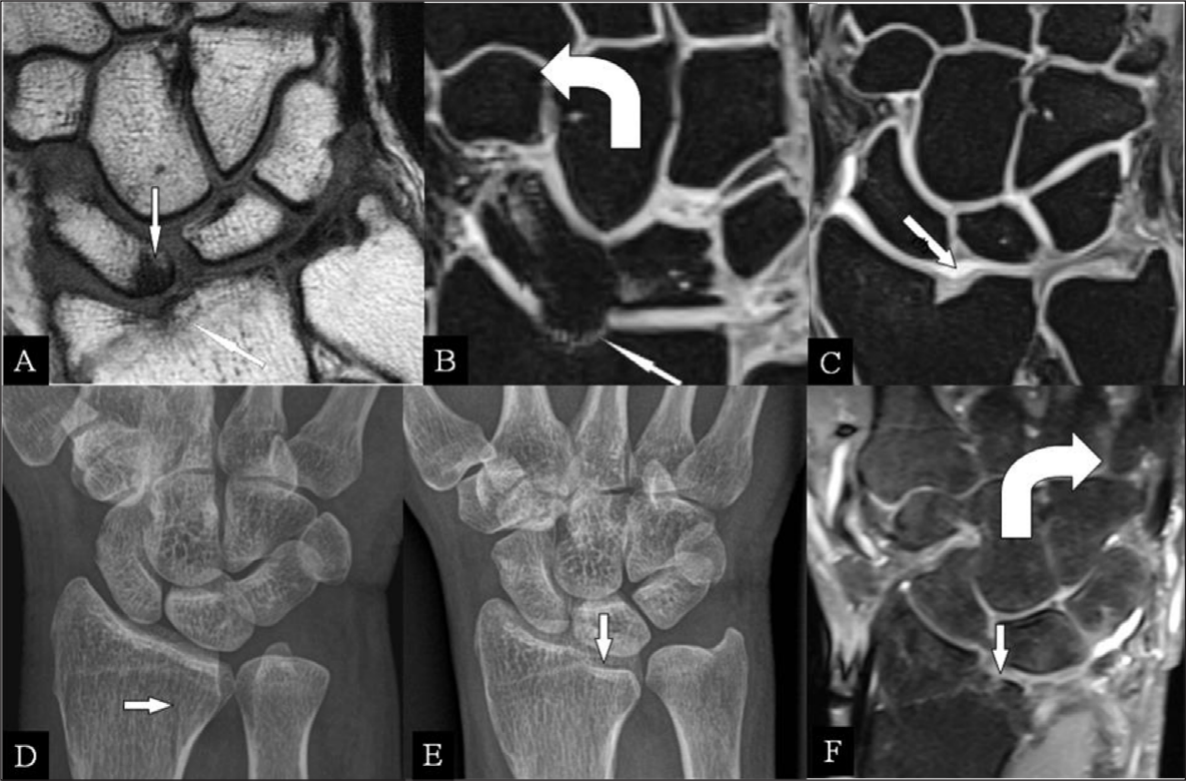
Radioscafoid and radiolunate abutment. **(A)** Coronal SE T1-WI; **(B, C)** Coronal 3D-GRE; **(D, E)** PA plain radiographs; and **(F)** Coronal SE PD-WI FS. (A) Sequela of an intra-articular fracture of the distal radial epiphysis with a residual step-off (oblique arrow) and marrow oedema (vertical arrow) at the proximal pole of the scaphoid bone. (B) Centrally, the cartilage is destroyed and the radial deviation is blocked. (C) Cartilage step-off in another patient. (D) The parasagittal intra-articular fracture was initially missed. (E) Consolidation with a depressed part of the articular surface. (F) Radiolunate abutment with blocked ulnar deviation.

Intra-articular fractures of the radius may heal with a residual step-off [6], seen on radiographs, CT, and MRI (Figure 1A–C). The radial deviation is limited. MRI illustrates the disappeared cartilage (Figure 1A and B) or the surface disruption (Figure 1C). In radial deviation (Figure 1B), bumping of the scaphoid against the prominent radial fossa zone causes repetitive impaction, resulting in BMO.

### Radiolunate abutment

Lunate bone impaction on its articular fossa may cause SAS. Parasagittal radial fractures (Figure 1D) need careful follow-up by radiographs or CT [6] in order to detect displacements, possibly causing SAS (Figure 1E and F) and limiting the ulnar deviation (Figure 1F).

### Radioulnar abutment

**Figure 2:**
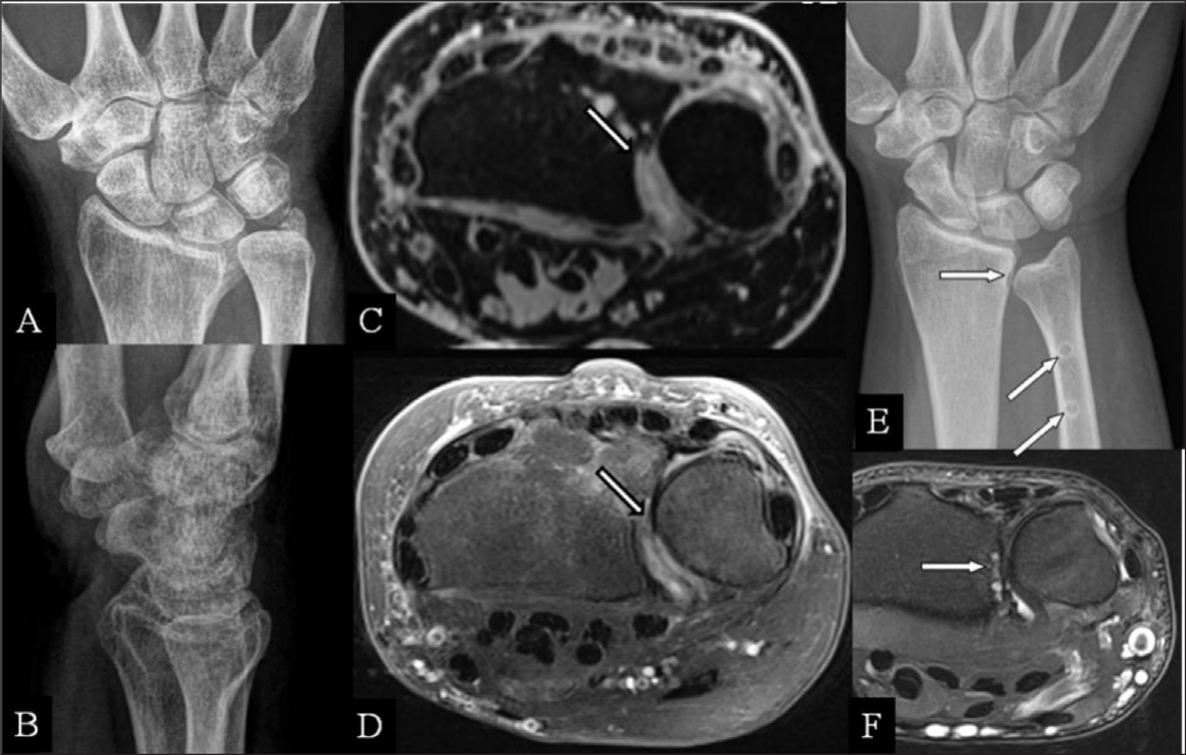
Radioulnar abutment and ulnar impingement. **(A, B)** PA and lateral plain radiographs; **(C)** Axial 3D-GRE; **(D)** SE T2-WI FS; **(E)** PA plain radiograph; and **(F)** Axial SE T2-WI FS. (A, B) Sequelae of a Pouteau-Colles fracture of the distal radial epiphysis. (C) Residual step-off at the radial sigmoid notch. (D) Destruction of the cartilage at the dorsal part of the sigmoid notch. (E) Excessive shortening (horizontal arrow) after surgery (oblique arrows). (F) subchondral erosions at the most proximal part of the radial sigmoid notch.

Distal radioulnar joint (DRUJ) fractures are difficult to evaluate on radiographs, particularly coronal sigmoid notch fractures (Figure 2A and B). Bony incongruity is better evaluated by (cone beam-)CT and a cartilage step-off (Figure 2C and D) by MRI. Pronation and supination may be hampered during the radial movement around the ulna [7]. Even minor deformities may cause severe dysfunction.

### Ulnar impingement

A relative ulnar shortening may appear after trauma, causing a DRUJ impingement (Figure 2E and F). Excessive ulnar shortening or resection may cause secondary impingement [8]. The appearance is equal to a congenital short ulna.

### Ulnar (intra)styloid abutment

**Figure 3:**
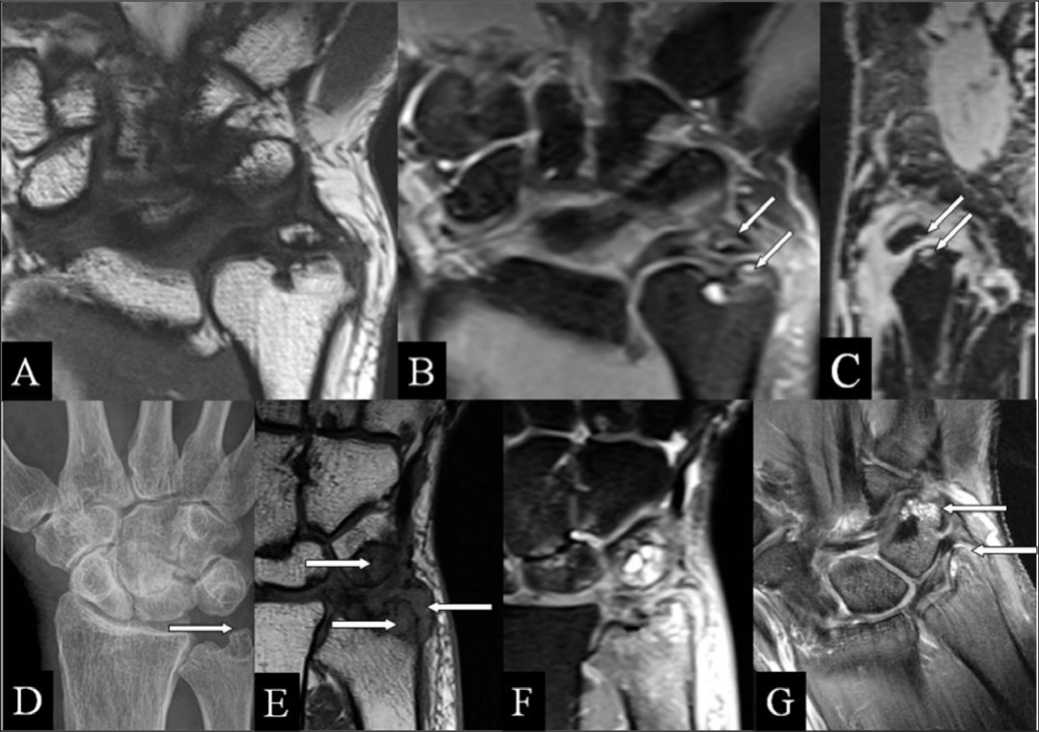
Ulnar (intra)styloid and stylotriquetral abutment. **(A, E)** Coronal SE T1-WI; **(B, F)** Coronal SE PD-WI FS; **(C)** Sagittal 3D-GRE; **(D)** PA plain radiograph; and **(G)** Coronal SE T1-WI FS with gadolinium. (A–C) Neoarticulation in the center of the ulnar styloid process, surrounding marrow oedema, (B) and juxta-articular cysts (arrows) (B, C). (D–F) Stylotriquetral abutment with flattening of the tip of the styloid process (D), bone marrow oedema and synovitis (E, F), and contrast enhancement of the marrow oedema and the synovitis (G).

Styloid process fractures may fail to heal, resulting into fragmentation and collision during ulnar deviation. MRI highlights neo-articulation and BMO (Figure 3A–C). The triangular fibrocartilage complex (TFCC) ulnar insertion (fovea and tip) in relation to these fragments is depicted (Figure 3B). Basal fractures may lead to DRUJ instability [9,10]. Ultrasound or MRI performed in pronation and supination may confirm a dynamic extensor carpi ulnaris tendon dislocation (empty sulcus sign).

### Stylo-triquetral abutment

Due to styloid process length increase, impaction with the triquetral bone may appear (Figure 3D–G). As in the classical abutment, the Garcia-Elias index [11] may indicate the risk of SAS development. Surrounding synovitis is frequent (prestyloidal synovitis).

### Ulnolunate and/or ulnotriquetral abutment

**Figure 4:**
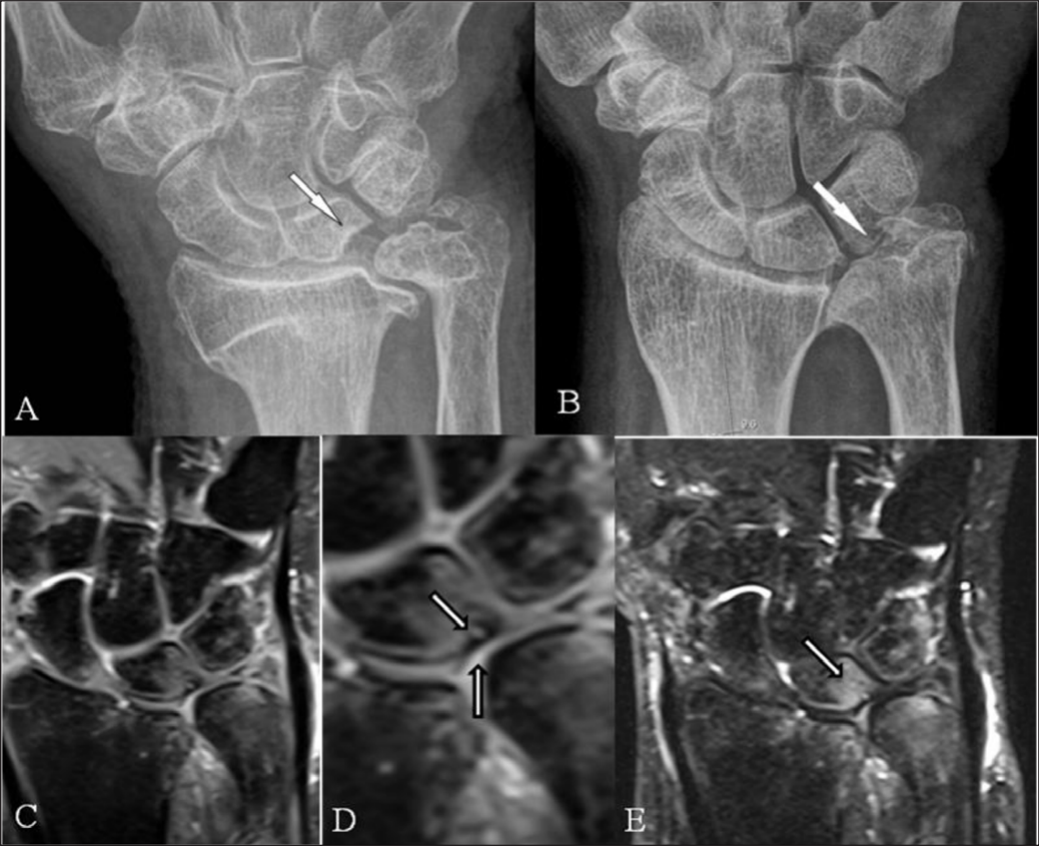
Ulnolunate and/or ulnotriquetral abutment. **(A, B)** PA plain radiographs; **(C, D)** Coronal SE PD-WI FS; and **(E)** Coronal SE T2-WI FS. (A) Ulnolunate abutment with a sclerotic defined impression at the ulnar side of the lunate bone. (B) Ulnotriquetral abutment with sclerotic bordered neoarticulation. (C–E) Ulnolunate abutment with chondromalacia at the ulnar border of the lunate bone (D, vertical arrow), subchondral cyst (D, oblique arrow), and bone marrow oedema centered at the ulnar side of the lunate bone (E).

In secondary positive ulnar variance, the latter abuts the lunate and/or triquetral bone and eventually leads to ulnar head deformation (Figure 4A–B). It may be associated with a TFCC tear. A lengthening of more than 3 mm may be symptomatic (Hulten criteria) [12]. TFCC lesions and BMO typically at the proximal-ulnar corner of the lunate bone are revealed early by MRI (Figure 4C–E).

### Ulnar translation with abutment

**Figure 5:**
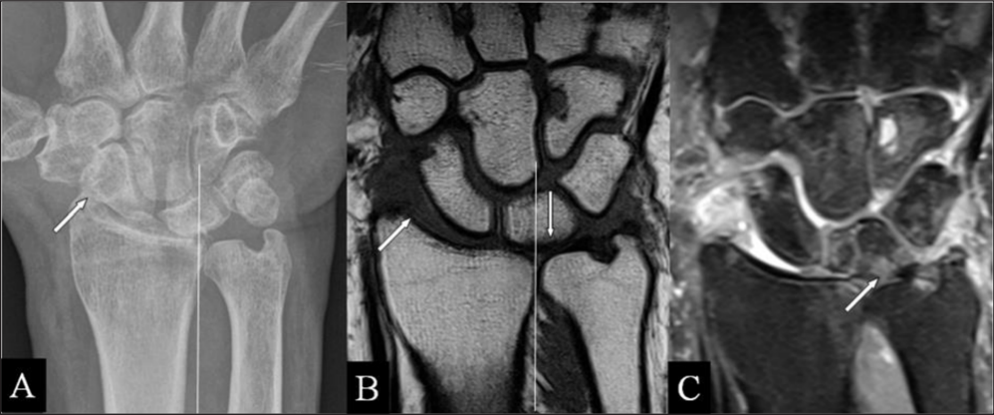
Ulnar translation with abutment. **(A)** PA plain radiograph; **(B)** Coronal SE T1-WI; and **(C)** Coronal SE PD-WI FS. (A–C) Lateral widening of the radioscaphoid joint (oblique arrow) and the lunate bone overlapping less than 50% with the corresponding radial articular fossa due to the ulnar translation of the carpus. (B, C) Cartilage destruction, oedema, and an accompanying lesion of the TFCC.

Extensive destruction of extrinsic ligaments leads to a proximal-ulnar carpal shift [13], creating a reversed status compared to ulnar abutment (ulna approaches carpus). The lateral widening of the radioscaphoid joint and the lunate position versus its corresponding articular fossa (less than 50% overlap in neutral position) are hallmarks on radiographs (Figure 5A). MRI presents the cartilage destruction, BMO, and eventual TFCC lesions (Figure 5B and C).

### (Intra)scaphoid abutment

**Figure 6:**
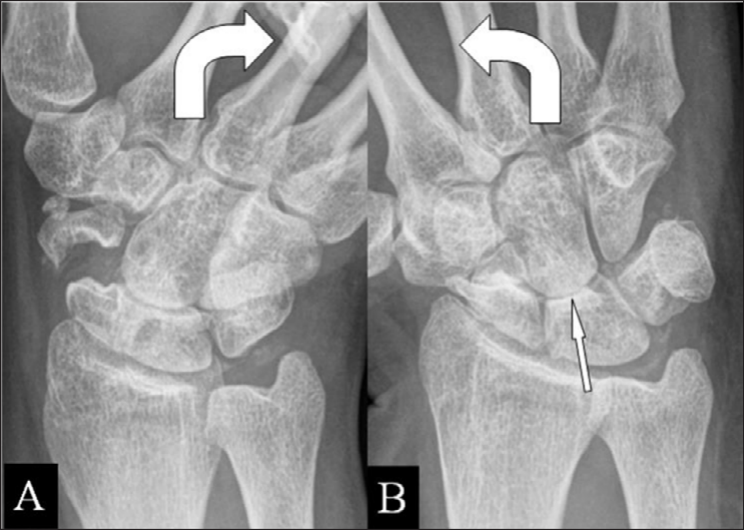
(Intra)scaphoid abutment. **(A, B)** PA plain mobility radiographs in ulnar and radial deviation. (A) Large diastasis in ulnar deviation between the scaphoid bone fragments. (B) Impaction of both fragments in radial deviation. Associated midcarpal osteoarthritis (arrow).

Fractures appear most frequently at the scaphoid waist [14]. Radial deviation gives an impaction at a pseudarthrosis. Contact zone deformation (Figure 6A and B) and mobility between the fragments are illustrated on radiographs. BMO and extrinsic ligamentous lesions are delineated by MRI.

### Lunar abutment

**Figure 7:**
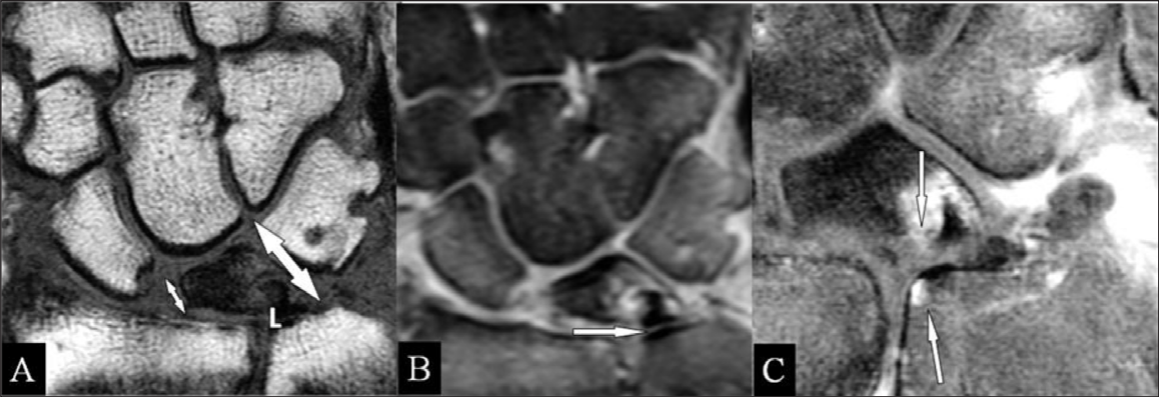
Lunar abutment. **(A)** Coronal SE T1-WI; **(B)** Coronal SE PD-WI FS; and **(C)** Coronal SE T1-WI FS with gadolinium. (A) Due to loss of height at the radial side, the ulnar side of the lunate bone (L) approaches the ulnar head. (B) Sclerotic borders at the contact zones (arrow), oedema at the ulnar corner of the lunate bone and TFCC tear. (C) Contrast enhancement in the zones of the kissing marrow oedema.

Avascular necrosis – probably due to chronic microtraumata – with deformation starts at the radial side [15]. Due to the height loss, the ulnar side approaches the ulna (reversed situation of ulnar abutment) (Figure 7A). The normal ulnar variance and the late appearance of sclerotic borders may be observed on radiographs. Early BMO and TFCC lesions call for MRI (Figure 7B and C).

### Scapholunar abutment

**Figure 8:**
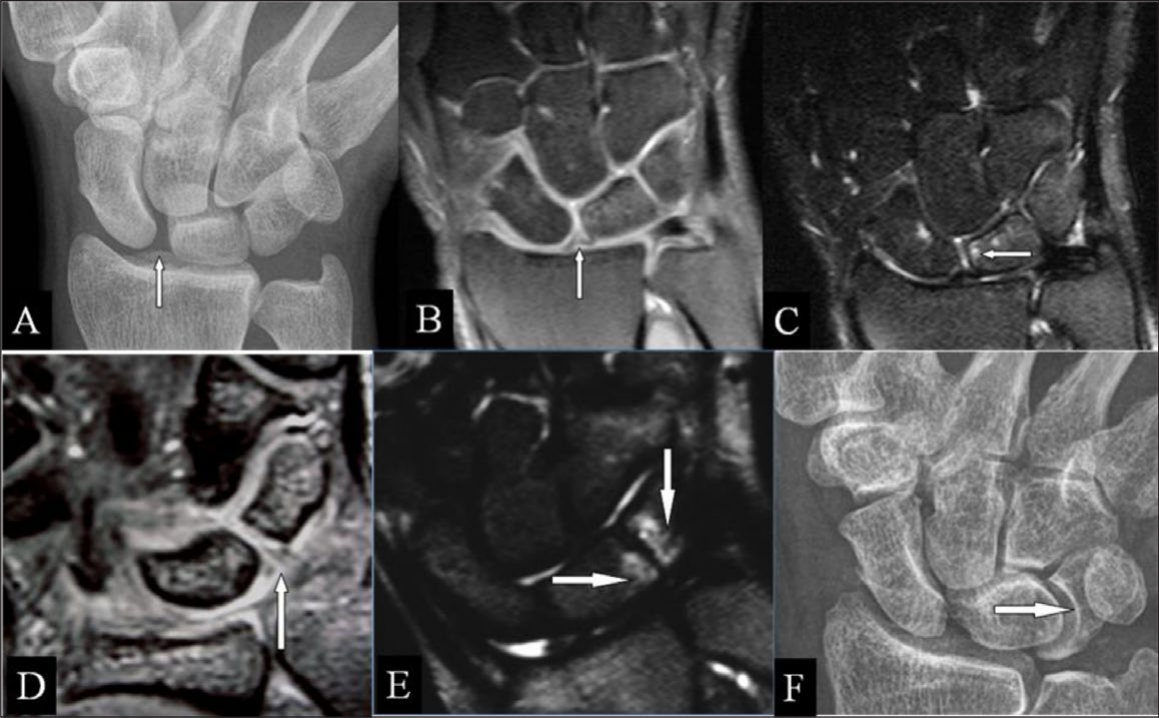
Scapholunar and lunotriquetral abutment. **(A)** PA plain radiograph; **(B)** Coronal SE PD-WI FS; **(C)** Coronal SE T2-WI FS; **(D)** Coronal 2D-GRE; **(E)** Coronal SE T2-WI FS; and **(F)** PA plain radiograph. (A) Widening of the scapholunate joint space on a Schneck I view. (B) Tear of the scapholunate ligament. (C) Juxta-articular subchondral band shaped marrow oedema. (D) Tear of the lunotriquetral ligament. (E) Juxta-articular band shaped kissing marrow oedema. (F) Massive deformation at the triquetral bone (other patient).

On radiographs (Figure 8A), a widened joint space (≥3 mm) [16] in rest or under stress (Schneck view) may be observed. MRI may show the ligamentous tear (Figure 8B) and the juxta-articular band shaped BMO (Figure 8C).

### Lunotriquetral abutment

MRI may also show this ligamentous tear (Figure 8D) and the band-shaped BMO (Figure 8E). Eventual concurrent extrinsic ligamentous lesions should be looked for, as it is questionable if this solitary lesion results in abnormal mobility [17]. Due to chronic impaction, massive deformation may appear on radiographs (Figure 8F).

### Hamatolunar abutment

**Figure 9:**
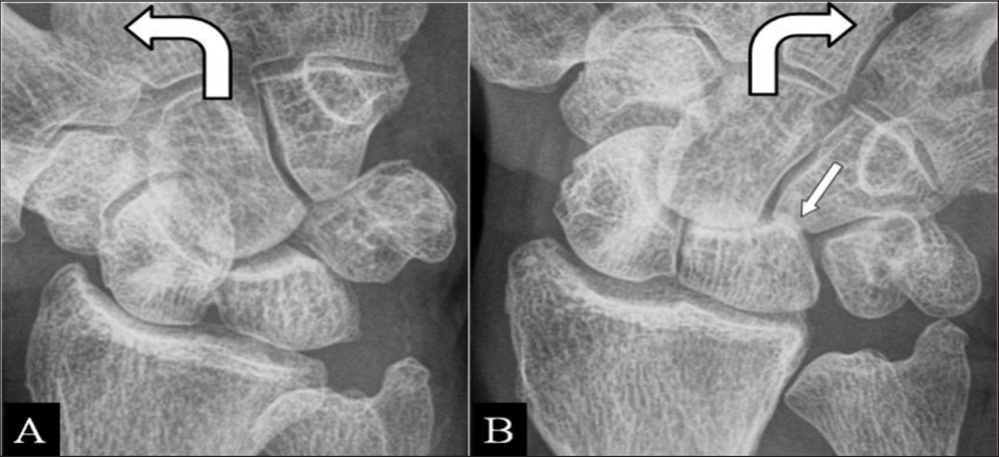
Hamatolunar abutment. **(A)** PA plain mobility radiographs in radial and ulnar deviation. (A, B) Step-off at the lunotriquetral joint of the first and second line of Gilula. **(B)** Secondary impaction of the hamate and lunate bone in ulnar deviation (oblique arrow).

Hamatolunar abutment is related to lunate bony variants (Viegas type II). However, a posttraumatic disturbance of the carpal row alignment (Gilula) [18] may induce SAS. Radiographs reveal the deformed area with abnormal contact during ulnar deviation (Figure 9A and B). The carpal line interruption and the pathologic bone motions are obvious on mobility radiographs.

### Carpal boss with abutment

**Figure 10:**
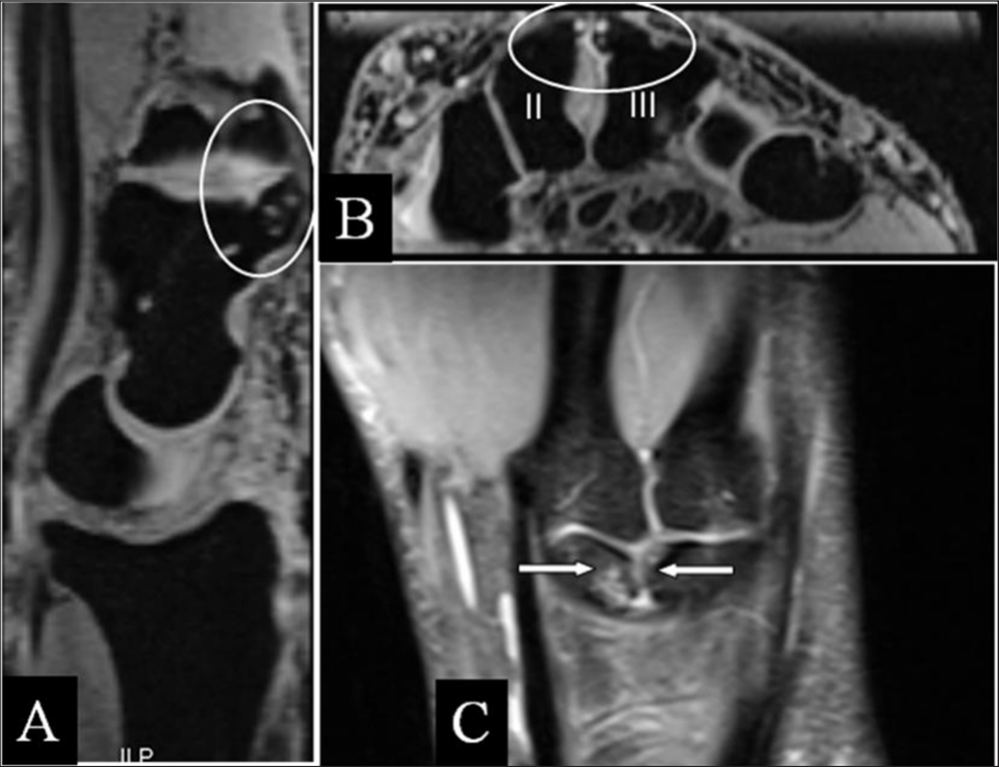
Carpal boss with abutment. **(A)** Sagittal 3D-GRE; **(B)** Coronal 3D-GRE; and **(C)** Coronal SE PD-WI FS. (A, B) Old posttraumatic deformation with subchondral cysts at the dorsum of the capitate bone (A) and the base of the second and third metacarpal bone (B) with misalignment around the quadrangular joint. (C) Juxta-articular kissing bone marrow oedema.

Quadrangular joint traumata may result in bony carpal boss [19]. Although the restricted motion, deformations induce juxtaarticular changes (Figure 10A–C). Due to superposition, radiographs are less useful and MRI may help to solve the clinical problem.

## Conclusion

A large variety of pathologies may cause SAS. This underscores the need for a thorough posttraumatic joint evaluation. Follow-up radiographs and MRI are mandatory in the presence of clinical symptoms. Concerns about the prognosis – certainly in expert or insurance-related files – should encourage detailed assessment, as even small lesions may be very functionally disabling.

## Important teaching points

Posttraumatic SAS may interfere with a large variety of normal daily activities, as the wrist is a crucial structure in the three-dimensional positioning of the hand. Some are fairly unknown and, due the complex anatomy of the wrist, a SAS is also often ignored or underappreciated.
